# Association of serum hemoglobin level with the risk of carotid plaque beyond metabolic abnormalities among asymptomatic adults without major adverse clinical events: a cross-sectional cohort study

**DOI:** 10.1186/s12872-021-01852-7

**Published:** 2021-03-02

**Authors:** Yunsuk Choi, Ki-Bum Won, Hyeon Hui Kang, Hyuk-Jae Change

**Affiliations:** 1 Division of Hematology and Oncology, Ulsan University Hospital, University of Ulsan College of Medicine, Ulsan, Republic of Korea; 2 Division of Cardiology, Ulsan University Hospital, University of Ulsan College of Medicine, Ulsan, Republic of Korea; 3 Division of Cardiology, Severance Cardiovascular Hospital, Yonsei University College of Medicine, Yonsei University Health System, 50-1 Yonsei-ro, Seodaemun-gu, Seoul, 03722 South Korea; 4 Division of Pulmonary, Critical Care and Sleep Medicine, College of Medicine, The Catholic University of Korea, Seoul, Republic of Korea; 5 Division of Pulmonary, Critical Care and Sleep Medicine, Ulsan University Hospital, University of Ulsan College of Medicine, 877 Bangeojinsunhwando-ro, Dong-gu, Ulsan, 44033 Republic of Korea

**Keywords:** Hemoglobin, Metabolic syndrome, Carotid plaque, Atherosclerosis

## Abstract

**Background:**

The serum hemoglobin (Hb) level is closely related to adverse clinical outcomes. However, data on the association of Hb levels with subclinical atherosclerosis beyond metabolic abnormalities are limited.

**Methods:**

This study evaluated the association among serum Hb level, metabolic syndrome (MetS), and the risk of carotid plaque formation in asymptomatic adults without a history of major adverse clinical events.

**Results:**

A total of 2560 participants (mean age: 60 ± 8 years, 32.9% men) were stratified into four groups based on Hb quartiles, as follows: ≤ 12.8 g/dL (group I), 12.9–13.6 g/dL (group II), 13.7–14.5 g/dL (group III), and ≥ 14.6 g/dL (group IV). The overall prevalence of MetS and carotid plaque was 37.2% and 33.4%, respectively. The prevalence of MetS increased with increasing Hb level (group I: 27.4% vs. group II: 35.9% vs. group III: 42.6% vs. group IV: 44.1%, *p* < 0.001). The prevalence of carotid plaque was 34.3%, 28.1%, 32.8%, and 39.5% in groups I, II, III, and IV, respectively. Univariate logistic regression analysis showed that MetS was associated with an increased risk of carotid plaque (odds ratio [OR] 1.568, 95% confidence interval [CI] 1.326–1.856, *p* < 0.001). Only group II showed a lower risk of carotid plaque than group I (OR 0.750, 95% CI 0.596–0.943, *p* = 0.014). Multiple logistic regression models showed consistent results after adjusting for clinical factors, including MetS and its individual components.

**Conclusion:**

Serum Hb level is associated with the risk of carotid plaque beyond MetS and its components in a relatively healthy adult population.

## Background

Low hemoglobin (Hb) levels are strongly associated with an increased risk of mortality in various clinical conditions, including acute coronary syndrome, heart failure, and chronic kidney disease [1–3]. In addition, recent studies have found that both low and high Hb levels are related to increased mortality, suggesting a U-shaped relationship [4–6]. This might imply the existence of a clinically beneficial level of Hb even within the normal Hb range. However, there is a paucity of data on this issue, especially on the relationship between Hb levels and subclinical atherosclerosis.

Metabolic syndrome (MetS) is a premorbid condition characterized by multiple metabolic disorders, with insulin resistance as a major component [7, 8]. The prevalence of MetS is rapidly increasing worldwide, and this entity affects approximately 31% of Korean adults [9, 10]. A number of previous studies have revealed that MetS is strongly associated with atherosclerotic cardiovascular (CV) disease and its related complications [11, 12]. In clinical practice, early detection of atherosclerosis is important for primary prevention in the asymptomatic general population [13]. Moreover, the treatment of atherosclerotic disease at an earlier stage and more precise patient selection are emphasized for primary prevention [14]. Previous landmark studies demonstrated that the assessment of subclinical atherosclerosis using carotid ultrasound, ankle-brachial index determination, and coronary calcium score calculation offered benefits for improved CV risk prediction beyond traditional risk factors [15–17]. In particular, the presence of carotid plaque is known to improve the prediction of new-onset CV disease based on the overall baseline CV risk status [15]. Therefore, the present study aimed to evaluate the association among serum Hb level, MetS, and the risk of carotid plaque formation, focusing on the comparison with the lowest category of Hb in asymptomatic adults without a history of major adverse clinical events.

## Methods

### Study design and participants

This cross-sectional investigation analyzed baseline data collected for a prospective cohort study. Briefly, a total of 2560 asymptomatic participants with no history of CV and cerebrovascular disease, neurological abnormalities, or malignancy took part in baseline health examinations in the Seoul area between April 2010 and November 2012 [11]. All participants were stratified into four groups based on Hb quartiles, as follows: ≤ 12.8 g/dL (group I), 12.9–13.6 g/dL (group II), 13.7–14.5 g/dL (group III), and ≥ 14.6 g/dL (group IV). Hypertension was defined as a systolic blood pressure (BP) of ≥ 140 mmHg and/or a diastolic BP of ≥ 90 mmHg, or use of anti-hypertensive medications. Hyperlipidemia was defined as a total cholesterol level of ≥ 240 mg/dL or treatment with lipid-lowering agents. Diabetes was defined as a fasting glucose level of ≥ 126 mg/dL or use of anti-diabetic medications. MetS was defined as present when three or more of the following criteria were satisfied: (a) systolic B* p* ≥ 130 mmHg or diastolic B* p* ≥ 85 mmHg, or use of anti-hypertensive medications; (b) abdominal obesity based on a waist circumference of ≥ 90 cm in men or ≥ 80 cm in women; (c) high-density lipoprotein cholesterol (HDL-C) level of < 40 mg/dL in men or < 50 mg/dL in women; (d) fasting triglycerides ≥ 150 mg/dL; and (e) fasting glucose ≥ 100 mg/dL or use of anti-diabetic medications [7].

### Measurements

**Fig. 1:**
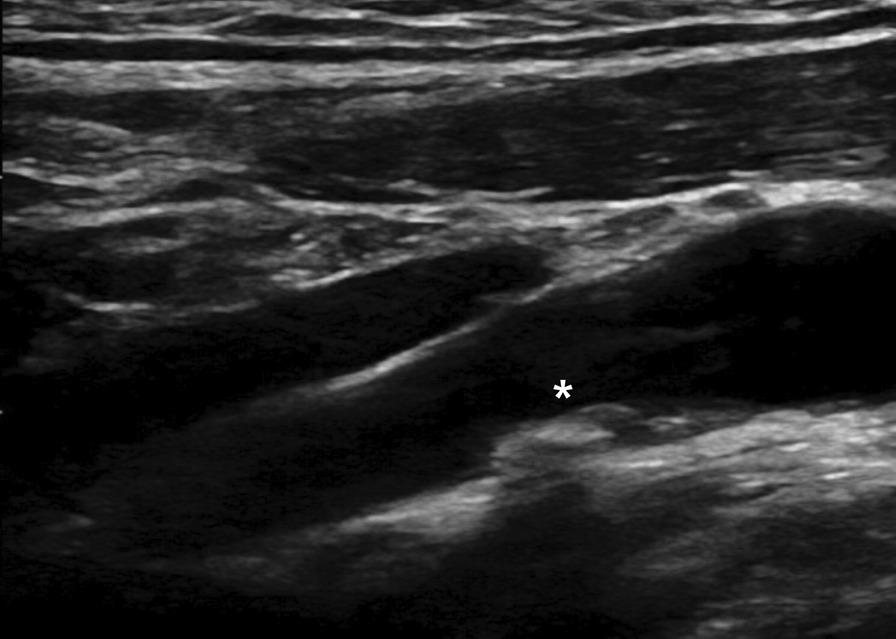
Representative image of a carotid plaque. The asterisk indicates a 1.0 × 0.3-cm plaque in the left internal carotid artery of a 54-year-old man

All blood samples were obtained after 8 h of fasting, and subsequently analyzed. Height and weight were measured with the participants wearing light clothing and no shoes. Body mass index was calculated as weight (kg) divided by the square of height (m^2^). Carotid ultrasound was performed with a high-resolution B-mode ultrasonography system (Acuson X300; Siemens, USA) with a transducer frequency of 13–15 MHz. Computer-assisted acquisition, processing, B-mode image storage, and calculation of intima-media thickness were performed using the Syngo Arterial Health Package (Siemens, USA). Automatic measurements of both common carotid arteries were performed at the far wall of the 1-cm segment distal to the carotid bulbs. Carotid plaque was defined as the presence of focal wall thickening ≥ 50% than that of the surrounding vessel wall or as a focal region with a carotid intima-media thickness of ≥ 1.5 mm, protruding into the lumen and distinct from the neighboring boundary [18, 19]. A representative image of a carotid plaque is presented in Fig. 1.

### Statistical analysis

Continuous variables are expressed as mean ± standard deviation. Categorical variables are presented as absolute values and proportions. The characteristics of participants across the four groups were compared using one-way analysis of variance or the Kruskal–Wallis test for continuous variables, as appropriate. The χ2 test or Fisher’s exact test was used for categorical variables, as appropriate. As the comparison of the prevalence of MetS and carotid plaque was performed based on the lowest Hb quartile, the p-value cutoff for statistical significance with Bonferroni correction was 0.017 for this analysis. Univariate logistic regression analysis was used to evaluate the association between clinical variables and the risk of carotid plaque. Multiple logistic regression models were used to evaluate the independent relationship between Hb levels and the risk of carotid plaque. The forced-entry method was used to enter independent variables into multiple regression models. All statistical analyses were performed using the Statistical Package for the Social Sciences software (version 19; SPSS, Chicago, IL, USA). A p value of < 0.05 was considered statistically significant for all analyses.

## Results

The baseline characteristics of the 2560 participants (mean age, 60 ± 8 years; 33% men) are presented in Table 1. The mean Hb levels were 12.2 ± 0.6, 13.3 ± 0.2, 14.1 ± 0.3, and 15.5 ± 0.7 g/dL in group I, II, III, and IV, respectively. The prevalence of male sex, hypertension, and smoking steadily increased with increasing Hb level. The levels of triglyceride and fasting glucose increased, whereas HDL-C levels decreased with increasing Hb levels.

**Table 1:** Baseline characteristics

		Quartiles of Hb				*p* value
	Total (n = 2,560)	I (lowest) (≤ 12.8 g/dL) (n = 647)	II (12.9–13.6 g/dL) (n = 725)	III (13.7–14.5 g/dL) (n = 585)	IV (highest) (≥ 14.6 g/dL) (n = 603)	
Age (years)	60.4 ± 7.9	60.8 ± 7.8	60.3 ± 7.3	60.9 ± 7.7	59.7 ± 8.7	0.029
Male sex, n (%)	842 (32.9)	56 (8.7)	82 (11.3)	193 (33.0)	511 (84.7)	< 0.001
BMI (kg/m^2^)	24.9 ± 3.0	24.3 ± 3.0	24.7 ± 2.9	25.1 ± 3.0	25.4 ± 2.9	< 0.001
Waist circumference (cm)	83.8 ± 8.5	80.9 ± 8.3	82.4 ± 8.3	84.4 ± 8.3	87.9 ± 7.5	< 0.001
Systolic BP (mmHg)	122.8 ± 15.1	119.2 ± 15.4	121.3 ± 14.7	124.0 ± 14.7	127.2 ± 14.5	< 0.001
Diastolic BP (mmHg)	73.9 ± 9.8	70.4 ± 9.7	72.2 ± 9.3	75.0 ± 8.9	78.7 ± 9.3	< 0.001
Hypertension, n (%)	1,273 (49.7)	297 (45.9)	341 (47.0)	299 (51.1)	336 (55.7)	0.002
Diabetes mellitus, n (%)	411 (16.1)	93 (14.4)	88 (12.1)	100 (17.1)	130 (21.6)	< 0.001
Hyperlipidemia, n (%)	938 (36.6)	268 (41.4)	275 (37.9)	212 (36.2)	183 (30.3)	0.001
Smoking, n (%)	695 (27.1)	51 (7.9)	85 (11.7)	170 (29.1)	389 (64.5)	< 0.001
Total cholesterol (mg/dL)	199.3 ± 36.2	198.2 ± 36.0	201.0 ± 37.2	199.9 ± 35.6	197.9 ± 35.9	0.364
Triglycerides (mg/dL)	129.0 ± 70.6	116.2 ± 61.9	120.5 ± 59.7	130.2 ± 66.8	151.9 ± 87.5	< 0.001
HDL-C (mg/dL)	54.4 ± 14.8	58.2 ± 14.9	55.8 ± 14.4	53.7 ± 14.5	49.2 ± 13.6	< 0.001
LDL-C (mg/dL)	121.6 ± 32.9	119.5 ± 32.6	123.2 ± 33.5	122.2 ± 32.5	121.3 ± 32.9	0.208
Fasting glucose (mg/dL)	101.3 ± 20.6	96.8 ± 15.1	98.5 ± 18.0	102.7 ± 21.3	108.1 ± 25.8	< 0.001
Creatinine (mg/dL)	0.79 ± 0.19	0.74 ± 0.20	0.73 ± 0.14	0.79 ± 0.18	0.91 ± 0.17	< 0.001

Values are presented as mean ± standard deviation or number (%)

BMI, body mass index; BP, blood pressure; Hb, hemoglobin; HDL-C, high-density lipoprotein cholesterol; LDL-C, low-density lipoprotein cholesterol; MetS, metabolic syndrome

**Fig. 2:**
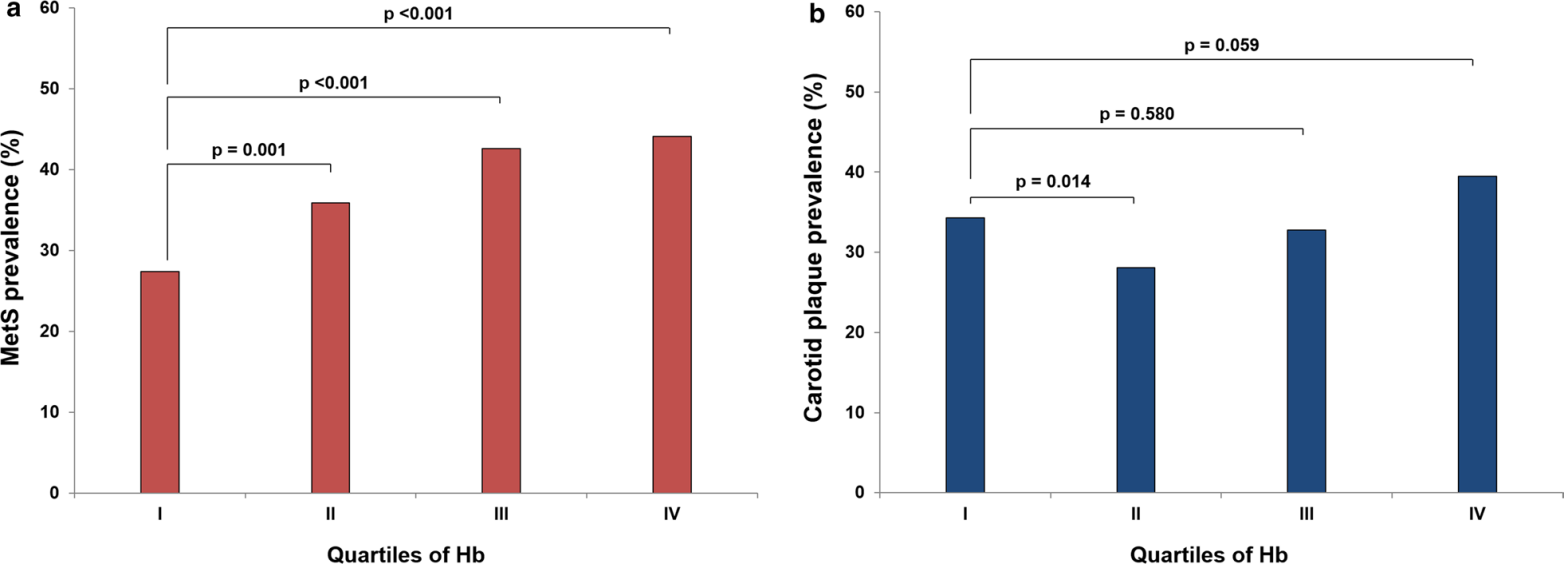
Prevalence of **a** MetS and **b** carotid plaque according to Hb quartiles. MetS = metabolic syndrome, Hb = hemoglobin

MetS was present in 952 (37.2%) participants. The prevalence of MetS was 27.4%, 35.9%, 42.6%, and 44.1% in groups I, II, III, and IV, respectively. Groups II, III, and IV showed a significantly higher prevalence of MetS than group I (Fig. 2a). Carotid plaque was observed in 856 (33.4%) participants. The prevalence of carotid plaque was 34.3%, 28.1%, 32.8%, and 39.5% in groups I, II, III, and IV, respectively. Only group II showed a significantly lower prevalence of carotid plaque than group I (*p* = 0.014) (Fig. 2b). Carotid plaque was more frequently observed in participants with MetS than in those without MetS (39.8% vs. 29.7%, *p* < 0.001).

In the univariate logistic regression analysis, age (odds ratio [OR] 1.090, 95% confidence interval [CI] 1.077–1.104), male sex (OR 1.826, 95% CI 1.538–2.168), MetS (OR 1.568, 95% CI 1.326–1.856), and smoking (OR 1.995, 95% CI 1.667–2.388) were associated with an increased risk of carotid plaque (all *p* < 0.01). Among the individual components of MetS, increased BP (OR 2.410, 95% CI 2.023–2.872, *p* < 0.001) and fasting glucose (OR 1.709, 95% CI 1.447–2.019, *p* < 0.001) were associated with an increased risk of carotid plaque. The risk of carotid plaque was significantly lower in group II than in group I (OR 0.750, 95% CI 0.596–0.943, *p* = 0.014), but was not significantly different between group I and groups III (OR 0.935, 95% CI 0.738–1.185, *p* = 0.580) and IV (OR 1.248, 95% CI 0.992–1.571, *p* = 0.590) (Table 2).

**Table 2:** Clinical variables and the risk of carotid plaque

	Carotid plaque	
	OR (95% CI)	*p* value
Age, per 1-year increase	1.090 (1.077–1.104)	< 0.001
Male sex	1.826 (1.538–2.168)	< 0.001
MetS	1.568 (1.326–1.856)	< 0.001
*Individual component of MetS*		
Increased BP	2.410 (2.023–2.872)	< 0.001
Increased waist circumference	1.047 (0.888–1.235)	0.582
Increased triglyceride	1.171 (0.975–1.406)	0.091
Decreased HDL-C	1.018 (0.852–1.216)	0.844
Increased fasting glucose	1.709 (1.447–2.019)	< 0.001
Smoking	1.995 (1.667–2.388)	< 0.001
Creatinine, per 1-mg/dL increase	5.304 (3.337–8.431)	< 0.001
*Categorical Hb groups*		
I	1	–
II	0.750 (0.596–0.943)	0.014
III	0.935 (0.738–1.185)	0.580
IV	1.248 (0.992–1.571)	0.590

BP, blood pressure; CI, confidence interval; Hb, hemoglobin; OR, odds ratio

The results of multiple logistic regression models for the association of Hb with the risk of carotid plaque are presented in Table 3. Compared with group I, only group II was consistently associated with a decreased risk of carotid plaque in all logistic regression models.

**Table 3:** Multiple logistic regression models for the association of Hb quartiles to the risk of carotid plaque

	Carotid plaque	
	OR (95% CI)	*p* value
*Model 1*		
I	1	–
II	0.762 (0.599–0.969)	0.026
III	0.814 (0.629–1.053)	0.117
IV	0.957 (0.707–1.296)	0.779
*Model 2*		
I	1	–
II	0.726 (0.569–0.928)	0.010
III	0.745 (0.572–0.972)	0.030
IV	0.832 (0.607–1.142)	0.255
*Model 3*		
I	1	–
II	0.747 (0.586–0.951)	0.018
III	0.772 (0.595–1.003)	0.053
IV	0.901 (0.662–1.225)	0.505

BP, blood pressure; CI, confidence interval; Hb, hemoglobin; HDL-C, high-density lipoprotein cholesterol; OR, odds ratio

Model 1: adjusted for age and sex

Model 2: adjusted for age, sex, systolic and diastolic blood pressure, waist circumference, and the level of triglyceride, HDL-C, and fasting glucose

Model 3: adjusted for age, sex, MetS, the level of creatinine, and smoking

## Discussion

In the present cohort of relatively healthy adults without a history of major adverse events, we investigated the beneficial range of Hb with respect to the risk of carotid plaque compared with the lowest category of Hb level, after adjusting for MetS and its individual components.

Low Hb level is a well-established risk factor for CV disease [20]. Furthermore, it is a substantial predictor of adverse clinical outcomes, independent of the CV risk status. Kalra et al. reported that a low Hb level was an independent predictor of mortality, CV events, and major bleeding in 21,829 patients with stable coronary artery disease [21]. Similar results have been reported in patients with acute coronary syndrome [1, 22, 23] and heart failure [24–26]. However, Tanne et al. observed that the association between the Hb level on admission and mortality was not linear and the risk of mortality increased at both extremes of Hb levels in patients with acute stroke [4]. Zakai et al. demonstrated that low and high Hb levels were independently associated with increased mortality in a prospective cohort study with 11.2 years of follow-up among 5888 community-dwelling men and women aged ≥ 65 years [5]. Kabat et al. showed a similar result in a large cohort of postmenopausal women [6]. Under conditions of high Hb levels, blood viscosity increases with elevated peripheral resistance and diminished cardiac output. An increase in blood viscosity affects coronary, cerebral, and peripheral blood flow as well as perfusion [27–30]. In addition, high Hb levels could stimulate atherogenesis through erythrocyte aggregation, leading to platelet aggregation and adhesion to the arterial wall [30–32]. AMORIS (Apolipoprotein MOrtality RISk Study), a study in 114,159 healthy men and women by Holme et al., suggested that high Hb levels are a risk factor for major atherosclerotic CV events [33]. These results suggest that there may be a clinically beneficial level of Hb even within the normal Hb range. However, there is a paucity of data on the association between serum Hb levels and the risk of subclinical atherosclerosis, especially in the healthy general population.

MetS is an important predictive factor for the development of diabetes and atherosclerotic CV disease. Initially, we identified that (a) the prevalence of MetS steadily increased with increasing Hb level and (b) MetS was significantly associated with an increased risk of carotid plaque. Given the relevance of Hb to oxygen-carrying capacity, oxidative stress, inflammatory processes, and blood viscosity, we hypothesized that a beneficial range of Hb for the risk of subclinical atherosclerosis compared with the lowest category of Hb level could be present in a relatively healthy adult population. This study utilized carotid plaque as a subclinical atherosclerotic parameter because of its incremental value in predicting CV events irrespective of the baseline CV risk status [15]. Interestingly, participants in group II were found to have a significantly lower risk of carotid plaque than those in group I after adjusting for MetS. Although the prevalence of carotid plaque in group IV was significantly higher than that in group III (39.5% vs. 32.8%, *p* = 0.017), no significant difference was observed between groups III and IV for the risk of carotid plaque after adjusting for MetS and its components. Considering that data on the optimal levels of Hb for reducing the risk of subclinical atherosclerosis in primary prevention have been limited, our results could provide evidence for this issue in a relatively healthy adult population.

This study had some limitations. First, all participants voluntarily participated in a general health examination. Therefore, selection bias might be present. Second, the study population was restricted to Korean participants, which may limit generalization. Third, we could not evaluate the significance of anemia for the risk of carotid plaque because of the extremely low prevalence of anemia, as only 23 (0.9%) participants had serum Hb levels < 11.0 g/dL. This might be related to the fact that our study was performed in asymptomatic relatively healthy adults who had no history of major adverse clinical events. Fourth, we did not perform subgroup analysis according to age categories and sex because the participants in this cohort study were relatively old and predominantly women. Given the variability in the distribution of MetS and Hb according to sex (Additional file 1: Fig. 1), it might be better to stratify the data; however, stratification was not feasible in the present study because of both the insufficient sample size and the skewed sex distribution. Further investigation with a larger sample size is necessary to identify the association of Hb with atherosclerosis, focusing on sex differences. Fifth, the design of this cohort study was cross-sectional. Thus, a longitudinal assessment related to the impact of Hb on subclinical atherosclerosis is necessary. Sixth, despite the significant association between obstructive sleep apnea and serum Hb levels [34], we did not consider this issue because of the paucity of data from the cohort registry. Seventh, despite the well-established reproducibility of carotid plaque measurement [35], this study was conducted without checking the interobserver agreement of carotid plaque measurements. Finally, we did not evaluate other environmental risk factors and the potential roles of exercise or diet in subclinical atherosclerosis. Despite these limitations, the present study is unique in that we identified the association of serum Hb level with the risk of subclinical atherosclerosis reflected in carotid plaque beyond MetS and its components in asymptomatic adults who had no history of major adverse clinical events.

## Conclusions

Serum Hb level is significantly related to the risk of carotid plaque after adjusting for metabolic abnormalities in relatively healthy Korean adults.

## Supplementary Information

**Additional file 1.** **Supplementary Fig. 1.** Sex difference in distribution of (A) MetS and (B) carotid plaque according to Hb levels. MetS = metabolic syndrome, Hb = hemoglobin.

## Data Availability

The datasets used and analyzed in the current study are available from the corresponding author on reasonable request.

## Abbreviations

BMI: Body mass index
CI: Confidence interval
CV: Cardiovascular
DBP: Diastolic blood pressure
Hb: Hemoglobin
HDL-C: High-density lipoprotein cholesterol
LDL-C: Low-density lipoprotein cholesterol
MetS: Metabolic syndrome
OR: Odds ratio
SBP: Systolic blood pressure
