# Supplementary figures and images for: Association of serum hemoglobin level with the risk of carotid plaque beyond metabolic abnormalities among asymptomatic adults without major adverse clinical events: a cross-sectional cohort study
Source: BMC Cardiovasc Disord. 2021 Mar 2;21:35. doi: 10.1186/s12872-021-01852-7 (PMC7923602; doi:10.1186/s12872-021-01852-7)

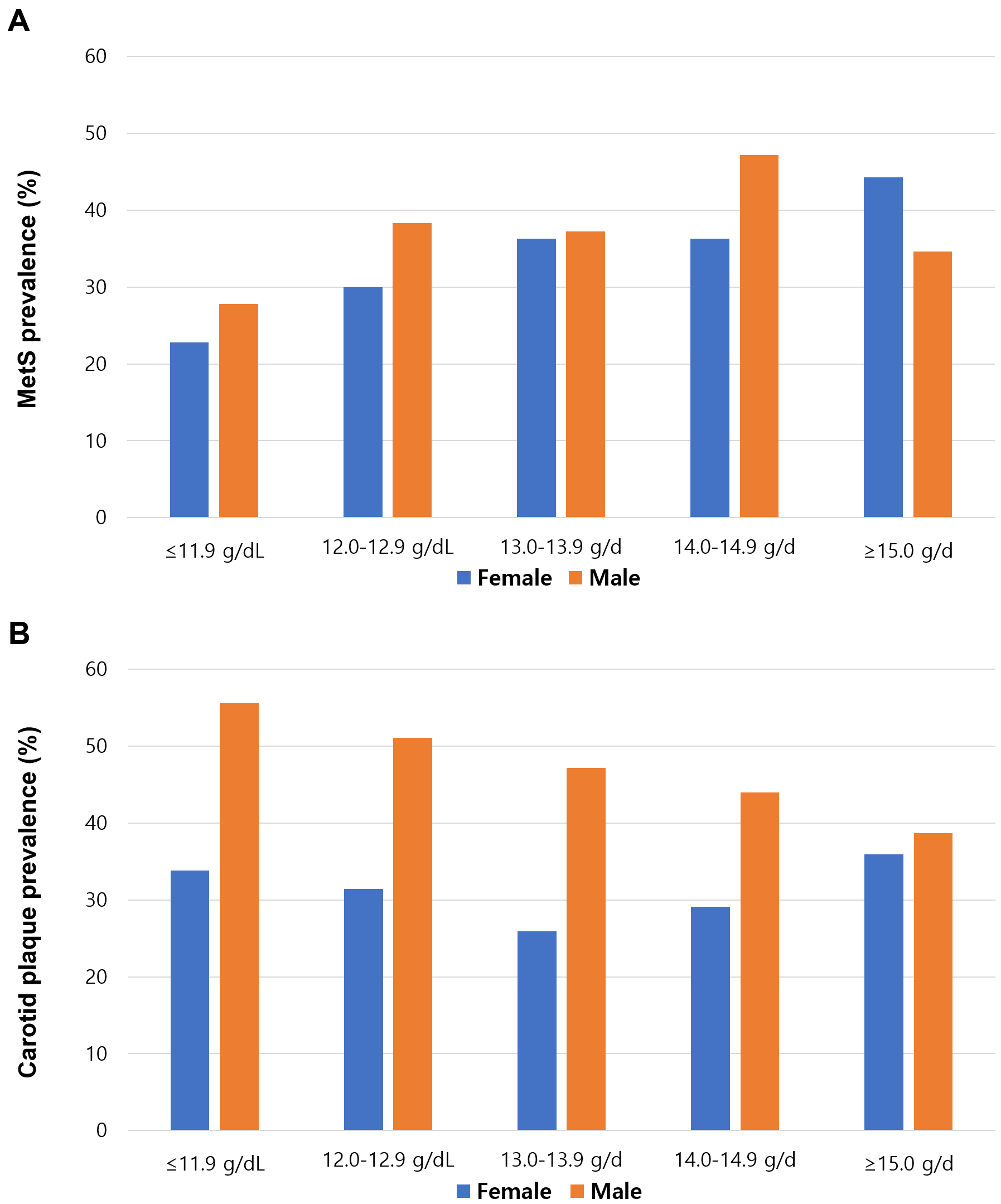

Supplement: Supplementary file 1 — Additional file 1. Supplementary Fig. 1. Sex difference in distribution of (A) MetS and (B) carotid plaque according to Hb levels. MetS = metabolic syndrome, Hb = hemoglobin. [file 12872_2021_1852_MOESM1_ESM.tif]
